# The CCT chaperonin is a context-dependent regulator of RNA-binding protein phase transitions

**DOI:** 10.17912/micropub.biology.002420

**Published:** 2026-09-25

**Authors:** Mingze Gao, Corrin C. Hays, Jennifer A. Schisa

**Affiliations:** 1 Central Michigan University, Mount Pleasant, MI, United States

## Abstract

RNA-binding proteins (RBPs) undergo regulated phase transitions during oogenesis that are critical for maternal mRNA regulation. In *C. elegans* , the CCT chaperonin prevents ectopic condensation of RBPs in maturing oocytes, including MEX-3 and CAR-1 . Here, we show that CCT differentially affects oocyte RBP condensation depending on protein identity and cellular context. Depletion of CCT did not cause ectopic condensation of the P-granule proteins GLH-1 and PGL-1 . During heat stress, CCT depletion reduced MEX-3 and CAR-1 condensation, opposite from the phenotype in normally maturing oocytes. These findings reveal CCT as a context-dependent regulator of RBP phase transitions in oocytes.

**Figure 1. The CCT chaperonin regulates RNA-binding protein phase transitions in oocytes in an RBP- and context-dependent manner:** A) Confocal images after individually depleting CCT subunits by RNAi in GLH-1 ::GFP and PGL-1 ::GFP strains. The negative control is RNAi of *lacZ.* The oocytes are numbered here, and in all panels, where -1 refers to the most proximal oocyte which will be fertilized next. B) Quantitation of the amount of GLH-1 condensed into granules in the -2 to -5 oocytes using ImageJ particle analysis. C) Quantitation of the amount of PGL-1 condensed into granules in the -2 to -5 oocytes using ImageJ particle analysis. D) Confocal images of oocytes after individually depleting CCT subunits by RNAi and exposing GFP:: MEX-3 worms to 34°C for 2 hours. E) Quantitation of the amount of condensed MEX-3 in the -2 to -4 oocytes using ImageJ skewness analysis. F) Confocal images of GFP:: CAR-1 germlines after depleting *cct-2* by RNAi and exposure to 34°C for 2 hours. The loop region of the germline is indicated by the dotted box. G) Quantitation of the amount of condensed CAR-1 in the -2 to -4 oocytes using ImageJ skewness analysis. Statistical significance was determined using the Kruskal-Wallis or Mann-Whitney U test. ns is not significant, *p<0.05, **p< 0.01, ****p< 0.0001. Scale bars are 10 microns.

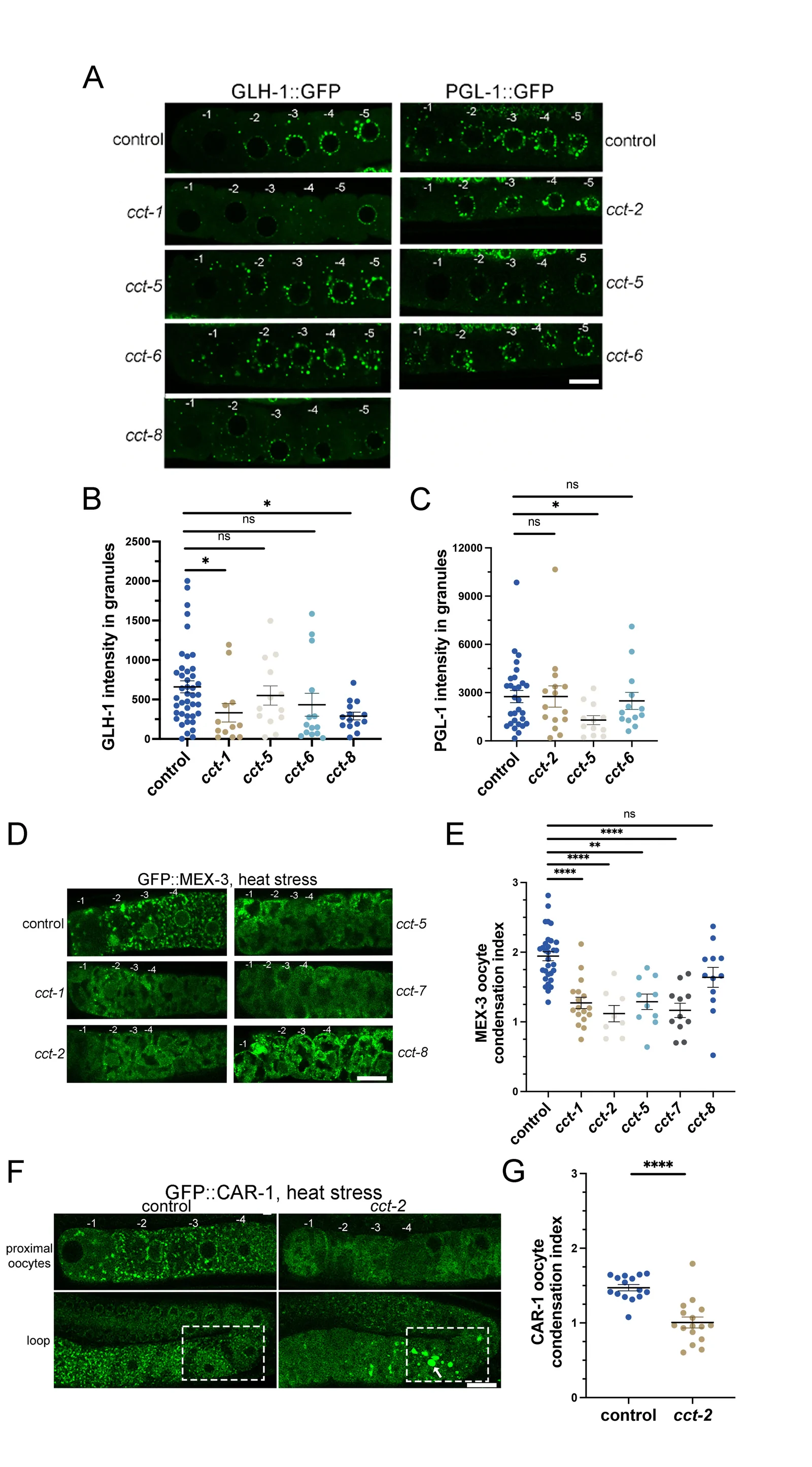



## Description

The regulation of maternal mRNAs by RNA-binding proteins (RBPs) during oogenesis is essential to produce viable gametes. Many oogenic RBPs undergo regulated phase transitions that are critical for their function. In many species, disruption of normally condensed or decondensed RBP phases alters mRNA metabolism and causes developmental defects (Cheng et al., 2022; Bose et al., 2022). In the *C. elegans* germline, some RBPs adopt a highly condensed phase and are concentrated in P-granules (e.g. PGL-1 , GLH-1 ), whereas others, including MEX-3 and LIN-41 , are largely dispersed or decondensed (Reviewed in Schisa, 2012).

We and others have identified regulators of RBP phase transitions in *C. elegans* oocytes, including the chaperonin-containing tailless complex polypeptide 1 (CCT) chaperonin (Hubstenberger et al., 2015; Wood et al., 2016; Elaswad et al., 2024). Individual depletion of seven of the eight CCT subunits results in ectopic MEX-3 condensates in maturing oocytes of young hermaphrodites, indicating that the CCT chaperonin is required to maintain MEX-3 in a decondensed phase. The CCT chaperonin also prevents ectopic condensation of three additional RBPs, CAR-1 , LIN-41 , and OMA-1 ; therefore, it is not specific to MEX-3 . FRAP analyses show that MEX-3 remains largely mobile within the ectopic condensates, suggesting they are not simply unfolded aggregates of MEX-3 (Elaswad et al., 2024). Thus, MEX-3 itself is unlikely to be a direct substrate of CCT. However, the mechanism by which the CCT chaperonin prevents condensation of RBPs remains unknown. In this study we asked if the CCT chaperonin similarly modulates phase transitions of P-granule proteins in maturing oocytes. We also investigated whether the CCT chaperonin regulates phase transitions of MEX-3 and CAR-1 during heat stress, a condition that induces phase transitions of several RBPs (Jud et al., 2008; Elaswad et al., 2022b).

To determine if the CCT chaperonin regulates phase transitions of P-granule proteins during oogenesis, we first depleted individual CCT subunits by RNAi in a GLH-1 ::GFP strain. In the *lacZ* negative control, GLH-1 was detected in both condensed P-granules and at low levels in a decondensed state in the oocyte cytosol ( Fig. 1A ). After individual depletion of four CCT subunits, the amount of GLH-1 condensed into granules in the -2 to -5 oocytes was either unchanged or modestly decreased relative to the control ( Fig. 1A, B). Although the amount of GLH-1 in granules varied somewhat across trials, we observed no consistent increase in GLH-1 condensation. This result contrasts with the ectopic condensation of several RBPs following CCT depletion, including in experiments performed in parallel which suggests the RNAi was at least partially effective (Elaswad et al., 2024). We next asked if CCT regulates PGL-1 condensation in oocytes. After individual depletion of three CCT subunits, the amount of PGL-1 in granules was either unchanged or modestly decreased relative to the *lacZ* negative control ( Fig. 1A, C). Together, these results indicate that the CCT chaperonin is not required to prevent ectopic condensation of GLH-1 and PGL-1 in maturing oocytes. Instead, the modest decreases in condensation raise the possibility that CCT contributes to P-granule condensation. If so, this effect is most likely indirect. Loss of CCT function is expected to disrupt folding of its substrates, and unfolded proteins often aggregate (Dunn et al., 2001), whereas we observed decreased condensation or aggregation of GLH-1 and PGL-1 . These experiments demonstrate that the requirement for the CCT chaperonin in preventing ectopic RBP condensation in oocytes is selective rather than universal.

Molecular chaperones play critical roles in maintaining protein homeostasis during cellular stresses, and MEX-3 condenses into large granules in oocytes during heat-stress (Jud et al. 2008; Koga et al. 2011; Elaswad et al. 2022b). Therefore, we investigated if CCT regulates MEX-3 condensation during heat stress as it does under normal developmental conditions. In the *lacZ* control worms exposed to heat stress, MEX-3 condensed into large granules in oocytes, as expected ( Figure 1D ). In contrast, after individual depletion of five *cct* subunits, we detected fewer and smaller MEX-3 granules in the oocytes. Moreover, the level of diffuse MEX-3 in the cytosol appeared to be higher than in control oocytes ( Figure 1D ). Quantification showed the amount of condensed MEX-3 was significantly reduced in the -2 to -4 oocytes relative to the negative control following depletion of four of five subunits tested ( Fig. 1E ). These data indicate that the CCT chaperonin promotes MEX-3 condensation in heat-stressed oocytes. Thus, CCT has opposing effects on MEX-3 phase behavior depending on the cellular context: it prevents ectopic MEX-3 condensation in normally maturing oocytes but promotes MEX-3 condensation during heat stress.

To determine if the context-dependent effect of CCT chaperonin on MEX-3 phase transitions was specific to MEX-3 , we examined CAR-1 , another RBP whose condensation in maturing oocytes is normally inhibited by the CCT chaperonin (Elaswad et al., 2024). We first tested if CAR-1 condenses during heat-stress, and we detected strong CAR-1 condensation ( Fig. 1F ). After depletion of *cct-2 ,* we detected significantly less CAR-1 condensation in the -2 to -4 oocytes, while the level of diffuse CAR-1 in the cytosol appeared to be higher than in control oocytes of heat-stressed worms ( Fig. 1F, G). Interestingly, in all *cct-2* worms we also detected condensates of increased size near the loop of the germline and/or in the distal germline that were not detected in any control germlines (dotted box in Fig. 1F ; p<0.0001).

Taken together, these findings extend our understanding of the CCT chaperonin as a regulator of RBP phase transitions during development. First, the selectivity of the CCT chaperonin in preventing ectopic RBP condensation argues against a model in which the ectopic condensation of MEX-3 and other RBPs following CCT depletion results simply from broad disruption of oocyte morphology or organization. Second, our results suggest the CCT chaperonin may contribute to promoting condensation of P-granule proteins in oocytes. The modest decreases in GLH-1 and PGL-1 condensation are consistent with a previous genetic screen that identified CCT subunits as promoters of PGL-1 condensation in embryos (Updike and Strome, 2009). These observations suggest CCT may promote P-granule assembly or stability at multiple stages of development. Lastly, the role we uncovered for the CCT chaperonin in promoting the condensation of MEX-3 and CAR-1 in heat-stressed oocytes was a striking contrast to its role preventing condensation in maturing oocytes. Interestingly, the CCT chaperonin also promotes condensation of RBPs in arrested oocytes (Hubstenberger et al., 2015; Wood et al., 2016). Prolonged meiotic arrest and heat stress may therefore induce similar cellular states that alter how CCT, its substrates, or associated pathways modulate RBP condensation. Our findings suggest the CCT chaperonin is a context-dependent regulator of RBP phase transitions during oogenesis.

## Methods

RNAi

RNAi clones were obtained from the Source Bioscience RNAi library (Kamath and Ahringer, 2003). A plasmid with the bacterial *lacZ* gene was used as the negative control in all experiments. All gene identities were verified by sequencing (Elaswad et al., 2024). RNAi was performed by feeding L4-stage hermaphrodites for 35 hours at 20°C. RNAi plates were blinded before image collection and analysis.

Heat stress

Heat stress experiments were performed as in previous studies (Elaswad et al., 2022b). The Tokai Hit stage top incubator was used with the Nikon A1R confocal system. Worms were placed at 34°C for two hours. The worms were transferred to agarose pads for imaging within 2 minutes of being at room temperature. Imaging was conducted at 34°C and was completed within 10 minutes of mounting worms to avoid imaging-associated stress (Elaswad et al., 2022a).

Microscopy

Worms were mounted on 2% agarose pads in 6.25mM levamisole, or 2.5mM levamisole for heat stress experiments to minimize bursting. Images were collected within 10 minutes of mounting on agarose pads using a Nikon A1R confocal system and a 60x N.A. 1.2 water-immersion objective. 0.5 micrometer slices were collected. Midfocal confocal slices were assembled for figure panels using Adobe Photoshop.

Quantitative and Statistical Analyses

GPower 3.1 was used to conduct a power analysis to determine sample size. A minimum of three biological replicates were performed for each RNAi experiment. To determine the relative amount of protein condensed into granules in oocytes, either ImageJ Particle Analysis or ImageJ Skewness tools were used as indicated in the figure legend. We did not include the -1 oocyte to avoid any complications with active meiotic maturation. To determine statistical significance, Kruskal-Wallis tests with Dunn's correction (or Mann-Whitney test for panel 1G) were conducted using GraphPad Prism v.10.2.0. Data are presented as mean +/- SEM. To determine if the percent of worms with an increased size of CAR-1 condensates at the loop or in the distal germline was significantly different after depletion of *cct-2 ,* we qualitatively scored the phenotype in ten control and ten *cct-2 (RNAi)* worms and conducted a Fisher's exact test. P values < 0.05 were considered statistically significant.

## Reagents

**Table:** 

Strain	Genotype	Available from
DUP64	glh-1 (sam24[ glh-1 ::GFP::3xFLAG])	Updike lab
JH3269	pgl-1 ( ax3122 [ pgl-1 ::gfp])IV	CGC
DG4269	mex-3 ( tn1753 [gfp::3xflag:: mex-3 ]) III	CGC
OD61	unc-119 ( ed3 )III; aIs1595[ pie-1 ::GFP-TEV-Stag:: CAR-1 ; unc-119 (+)]	CGC
